# Essential updates to the surgical treatment of biliary tract cancer

**DOI:** 10.1002/ags3.12266

**Published:** 2019-05-22

**Authors:** Satoshi Matsukuma, Yukio Tokumitsu, Yoshitaro Shindo, Hiroto Matsui, Hiroaki Nagano

**Affiliations:** ^1^ Department of Gastroenterological, Breast and Endocrine Surgery Yamaguchi University Graduate School of Medicine Ube Japan

**Keywords:** adjuvant therapy, biliary tract cancer, cholangiocarcinoma, gallbladder cancer, surgical treatment

## Abstract

Biliary tract cancer, which includes intrahepatic cholangiocarcinoma, extrahepatic bile duct cancer, gallbladder cancer, and ampullary cancer, is an intractable disease with a dismal prognosis. Prognosis is particularly poor in cases involving vessels or lymph nodes. Hepatobiliary pancreatic surgeons worldwide have consistently focused on improving surgical treatment, perioperative management, and chemotherapy to improve the outcomes of these diseases. There has been significant progress even in the last 2 years (2017 and 2018), such as promising findings reported by studies on the optimal extent of surgical treatment and multi‐institutional randomized controlled trials on adjuvant chemotherapy. We overview the current trends and advancements made in surgical treatment in 2017 and 2018.

## 1 INTRODUCTION

Biliary tract cancer (BTC) arises from the epithelium of the biliary tree, from the periphery of the liver to Vater's papilla, and includes intrahepatic cholangiocarcinoma (ICC), bile duct cancer as represented by perihilar cholangiocarcinoma (PHCC), gallbladder cancer (GBC), and ampullary cancer. Surgical resection is the only curative treatment for BTC, and although the efficacy of chemotherapy has not been fully established, there have recently been some promising advancements. In this biannual review, we review essential updates to the treatment of BTC worldwide in the 2‐year period between 2017 and 2018. The 19 most important articles published in the last 2 years and selected according to impact factor offered in InCites Journal Citation Reports (https://jcr.clarivate.com/JCRJournalHomeAction.action) are summarized in Table 1, and randomized controlled trials (RCT) published in these periods are summarized in Table 2.

**Table 1:** Essential updates to the treatment of biliary tract cancer published in the 2‐y period between 2017 and 2018

First author	Disease	Study design	No. patients	Information
Aoki^1^	BTC	Retrospective	52	HPD using PBD, PVE and two‐stage pancreaticojejunostomy could be done safely with near‐zero mortality and acceptable long‐term outcome (5‐y OS: 44.5%)
Ebata^2^	PHCC	Retrospective	216	Resection for Bismuth type IV PHCC can be done safely with low mortality rate and provides favorable long‐term outcome in selected patients (5‐y OS for patients with pN0M0 disease: 53%)
Bird^3^	PHCC	Retrospective	116	Staging laparoscopy could be useful for detection of radiologically occult metastasis
Zhang^4^	ICC	International database	1084	Use of LND has increased over time in most countries
Kasumova^5^	GBC	US national database	6825	Extended cholecystectomy with adjuvant chemotherapy could improve outcome of patients with pT2/T3 GBC
Ethun^6^	GBC	USEBMC	207	Optimal time interval to additional resection for incidental GBC may be between 4 and 8 wks after the initial resection
Kishi^7^	GBC	Retrospective	259	PSPLN should be considered as regional nodes to be resected
Maeta^8^	DCC	Retrospective	453	PVR for DCC did not contribute to long‐term outcome
Yamamoto^9^	GBC	Retrospective	96	Major hepatectomy with PVR or PD may be an acceptable procedure for advanced GBC, especially in selected patients without liver metastasis or hepatic arterial invasion
Sugawara^10^	BTC	RCT	86	Giving 2‐day prophylactic antibiotics is sufficient for patients undergoing hepatectomy with EBDR
Watanabe^11^	BTC	Retrospective	225	Minimum %FLV limit in major hepatectomy with EBDR should be set at ≥45% in patients aged >69 y
Yamashita^12^	BTC	Retrospective	312	Non‐normalization after curative resection was associated with poorer survival
Raoof^13^	ICC	Retrospective	275	Development of prognostic score to predict survival after hepatectomy
Orimo^14^	ICC	Retrospective	104	Although non‐curative resection was more frequent in hilar‐type ICC than in peripheral‐type ICC for advanced disease, survival in curative resection cases was similar between hilar‐ and peripheral‐type ICC
Zhang^15^	ICC	Retrospective	933	Tumor size and number of tumors were associated with early (<24 mo) intrahepatic recurrence and only the presence of liver cirrhosis was associated with late intrahepatic recurrence (>24 mo)
Komaya^16^	DCC	Retrospective	389	Risk factors for recurrence after curative resection were perineural invasion, pancreatic invasion and positive nodal involvement, and survival could be stratified by the corresponding number of these three factors
Ethun^17^	PHCC	USEBMC	232	Overall survival of patients with PHCC <3 cm without lymph node metastasis who underwent LT (n = 41) was better than in those who underwent resection (n = 191)
Ebata^18^	BDC	RCT	225	Adjuvant chemotherapy with GEM failed to improve survival of patients with resected BDC compared to observation
Kobayashi^19^	BTC	RCT	70	Adjuvant chemotherapy with S‐1 might improve survival of patients after major hepatectomy compared to those with GEM

Abbreviations: BDC, bile duct cancer; BTC, biliary tract cancer; DCC, distal cholangiocarcinoma; EBDR, extrahepatic bile duct resection; FLV, functional liver volume; GBC, gallbladder cancer; GEM, gemcitabine; HPD, hepatopancreaticoduodenectomy; ICC, intrahepatic cholangiocarcinoma; LND, lymph node dissection; LT, liver transplantation; OS, overall survival; PBD, preoperative biliary drainage; PD, pancreaticoduodenectomy; PHCC, perihilar cholangiocarcinoma; PSPLN, posterior superior pancreaticoduodenal lymph nodes; PVE, portal vein embolization; PVR, portal vein resection; RCT, randomized controlled trial; USEBMC, United States Extrahepatic Biliary Malignancy Consortium.

**Table 2:** Randomized controlled trials published in the 2‐y period between 2017 and 2018

First author	No. patients	Objective	Comparison	Information
Sugawara^10^	BTC (n = 86)	Optimal duration of prophylactic antibiotics for patients undergoing major hepatectomy with EBDR	2‐d (n = 43)	Incidence of any infectious complications was similar between the two groups
			4‐d (n = 43)	
Yamamoto^20^	BTC (n = 41)	Optimal duration of prophylactic antibiotics for patients undergoing PD after PBD	1‐d CZOP (n = 40)	Incidence of infectious complications was lower in the 1‐d group
	PDAC (n = 37)		5‐d CZOP (n = 42)	
	Other (n = 4)			
Coelen^21^	PHCC (n‐54)	Incidence of severe drainage‐related complications	EBD (n = 27)	This study was prematurely closed because of drastically higher mortality in the PTBD group
			PTBD (n = 27)	
Ebata^18^	BDC (n = 225)	Efficacy of adjuvant GEM chemotherapy	GEM (n = 117)	Survival was not different between the two groups
			Observation (n = 108)	
Kobayashi^19^	BTC (n = 70)	Efficacy of adjuvant GEM and S‐1 after major hepatectomy	GEM (n = 35)	Survival of adjuvant S‐1 therapy group was superior to that of GEM group
			S‐1 (n = 35)	

Abbreviations: BTC, biliary tract cancer; CZOP, cefozopran; EBD, endoscopic biliary drainage; EBDR, extrahepatic bile duct resection; GEM, gemcitabine; PBD, preoperative biliary drainage; PD, pancreaticoduodenectomy; PDAC, pancreatic ductal adenocarcinoma; PHCC, perihilar cholangiocarcinoma; PTBD, percutaneous transhepatic biliary drainage.

## 2 SURGICAL TREATMENT

### 2.1 Surgical safety

In a report by the Japanese Society of Hepato‐Biliary‐Pancreatic Surgery (JSHBPS), 90‐day mortality rates of surgery for biliary diseases over the 4‐year period between 2012 and 2015 were higher than those for hepatic diseases or pancreatic diseases; for example, left hepatic trisectionectomy (10.3%), hepatopancreatectomy (HPD; 7.6%), hepatectomy with extrahepatic bile duct resection (4.6%), and right hepatic trisectionectomy (4.5%).^22^ A study from two institutes in Japan and the UK also showed a high mortality rate of trisectionectomy (90‐day mortality; right: 10.5%, left: 23.1%) for BTC.^23^ However, although the surgical outcomes of biliary diseases remain unsatisfactory, the 90‐day mortality rate of high‐level hepatobiliary pancreatic (HBP) surgery at board‐certified training institutions has decreased significantly in Japan since the establishment of a board certification system, from 2.1% in 2012 to 1.3% in 2015.^22^

Centralization of HBP surgery to regional tertiary centers, which has been introduced globally, has contributed to improvement of short‐ and long‐term outcomes of patients. However, the inconvenience of treatment referrals in this system can impose difficulty for patients who live far from the tertiary center, and subsequent delays in treatment can adversely affect patient outcomes as a result of tumor progression.^24^ In contrast, Amr et al^24^ reported that patients’ traveling distance to a tertiary center did not affect the interval between diagnosis of periampullary cancer (PC) and surgery, pathological stage, or the long‐term outcome of patients with PC. Those authors concluded that centralization of HBP surgical services can be implemented without imposing disadvantages in surgical outcome on patients for travel distance to the tertiary center or referral between hospitals.^24^

A hepato‐biliary‐pancreatic surgical research group from the University of Tokyo reported the safety of HPD with two‐stage pancreaticojejunostomy for BTC.^1^ They carried out HPD without pancreatic reconstruction and two‐stage pancreaticojejunostomy approximately 3 months after resection, achieving near‐zero mortality and acceptable long‐term outcomes (5‐year overall survival [OS]: 44.5%).^1^ Surgical outcomes for Bismuth type IV PHCC was reported by the Nagoya University group, who carried out 216 resections including 131 combined vascular resections; they reported a 41.7% morbidity rate of Clavien‐Dindo grade III or higher, a 1.9% 90‐day mortality rate, and a 5‐year OS and median survival time (MST) of 32.8% and 34.9 months, respectively.^2^

In Japan, the number of older patients with cancer who require resection has been increasing because of an increasingly aged population. A recent report from the Nagoya group showed that resection of PHCC could be carried out safely with acceptable long‐term outcomes even in octogenarians if resection was based on careful preoperative evaluations of comorbidity and decreased organ function.^25^

### 2.2 Optimal surgical procedure

#### 2.2.1 Perihilar cholangiocarcinoma

In a recent study, staging laparoscopy was carried out for 114 patients with radiologically resectable disease; 29 patients (64.4%) were detected among 45 patients with unresectable disease, including radiologically undetectable peritoneal metastases, locally advanced disease, and intrahepatic metastases, thus avoiding unnecessary laparotomy and insufficient resection.^3^

Hepatopancreatectomy carried out for BTC with extensive horizontal spreading between the hepatic hilum and the intrapancreatic bile duct is associated with a high risk of morbidity and mortality,^22^ and hepatic failure and postoperative pancreatic fistula are the most common and life‐threatening complications of this procedure. However, a study by Chiba et al^26^ reported that a modified technique of HPD with delayed dissection of the pancreatic parenchyma minimized peripancreatic saponification and prevented pancreatic fistula.

The Shimane University research group in Japan proposed a liver parenchyma transection‐first approach in hemihepatectomy with total caudate lobectomy which used a modified liver‐hanging maneuver as a safe and efficient procedure for reducing blood loss, liver failure, and mortality.^27^ A recent report described the efficacy of an intrahepatic approach to Glisson's sheath in left‐sided hepatectomy following transection of the hepatic parenchyma in which the bile duct was dissected to access the distal portion of the invaded right hepatic artery and/or portal vein; this approach was recommended when an extrahepatic approach to access the distal portions of the invaded vessels is not possible.^28^

#### 2.2.2 Intrahepatic cholangiocarcinoma

A recent study comparing perioperative and long‐term outcomes of patients with ICC who underwent major and minor hepatectomy showed that the incidence of postoperative complications and mortality was higher in major hepatectomy, whereas survival was similar between the two groups after propensity‐score matching (PSM).^29^ Similarly, another report evaluated perioperative and long‐term outcomes of patients with solitary ICC who underwent anatomical resection compared with the outcomes of patients who underwent non‐anatomical resection, and showed a higher postoperative complication rate in anatomical resection and similar long‐term outcomes between the two groups.^30^

Although lymph node (LN) metastasis is an extremely poor prognostic factor in ICC, the value of LN dissection (LND) for ICC remains controversial. As LN metastasis is a systemic disease that spreads through lymphatic drainage routes in multiple directions, some clinicians believe that systemic LND might merely be LN sampling. However, systemic LND could be acceptable under circumstances where preoperative imaging of LN metastasis is insufficient. Current trends in LND have been reported from two multi‐institutional databases in the USA^31^ and 15 major hepatobiliary centers in the USA, Europe, Australia and Asia.^4^ Although the proportion of patients who underwent LND was lower in Western countries^4^, ^31^ compared to Asian countries,^4^ the use of LND has increased over time in most countries.^4^, ^31^

#### 2.2.3 Gallbladder cancer

The Japanese guidelines recommend additional resection for patients with GBC invading the subserosal layer (T2) or deeper,^32^ whereas the American guidelines recommend additional resection for patients with T1b‐T3 disease.^33^ A recent international multicenter study of 237 patients with T1b GBC showed that the outcomes of simple cholecystectomy (SC) were similar to those of extended cholecystectomy (EC), indicating that EC may not be needed for T1b GBC.^34^ The superiority of EC to SC for patients with pT2/T3 GBC was confirmed in a study using the US National Cancer Data Base under the limiting condition of adjuvant therapy being carried out.^5^ In contrast, however, a different study reported that additional resection could not improve the survival of patients diagnosed with incidental T1b/T2 GBC, especially in the presence of residual disease.^35^

Little is known about the optimal time interval to additional resection for incidental GBC. Ethun et al^6^ classified patients into three groups according to the time interval from initial cholecystectomy to reoperation and analyzed the outcomes of patients using the US Extrahepatic Biliary Malignancy Consortium database. That study showed that patients who underwent reoperation between 4 and 8 weeks had the longest median OS (40.4 months) compared with those who underwent reoperation within <4 weeks (median OS, 17.4 months) or after more than 8 weeks (median OS, 22.4 months).^6^ However, the number of patients in each group was relatively low, and further investigation is needed.

The cystic duct node (LN station 12c) is known as the initial site of lymphatic metastasis from GBC and is frequently removed in the initial cholecystectomy of lesions suspected to be GBC. According to a recent report, the status of the cystic duct node could predict the hepatic pedicle node status but could not predict more advanced LN status; moreover, the outcome of patients with metastasis in the cystic duct node only without residual cancer at additional resection was similar to the outcome of those with negative nodes.^36^ The authors of that study recommended D2 LND at additional resection regardless of cystic duct node status.^36^

Posterosuperior pancreatic head LN (PSPLN, LN station #13a) are included in regional LN only in the staging system of the JSHBPS but not in the American Joint Committee on Cancer (AJCC)^37^ or Union for International Cancer Control (UICC)^38^ staging systems. Recently, Sakata et al^39^ reported that the metastatic rate to PSPLN was 12.8% with a 5‐year survival rate of 31.6% in patients with positive nodes. The outcome after resection of patients with positive distant nodes in PSPLN only was significantly better than that in patients with positive distant nodes beyond the PSPLN (5‐year survival, 55.6% vs 15.0%, *P* = 0.046), while the outcome of the former group was comparable with that of patients with regional nodal disease.^39^ Similarly, Kishi et al^7^ classified patients into three groups: patients with nodal metastases limited to the hepatoduodenal ligament or common hepatic artery (Na); extending to the PSPLN (Nb); or in nodes along the celiac axis or superior mesenteric vessels (Nc). The authors reported that 5‐year disease‐specific survival (DSS) was comparable between patients with NaM0 and those with NbM0 disease, whereas those with NcM0 had worse outcomes than those with NbM0 and comparable outcomes to those with distant metastases.^7^ Moreover, the 5‐year DSS was comparable between patients who underwent pancreaticoduodenectomy (PD) and patients who underwent dissection of PSPLN without PD.^7^ These data showed that PSPLN should be considered as regional LN to be resected for GBC, and that PD is not needed for dissection of PSPLN only.

It is generally accepted that patients with hepatic‐sided T2 GBC have worse outcomes than those with peritoneal‐sided GBC; however, whether liver resection can improve the outcomes of patients with hepatic‐sided T2 GBC remains controversial. Recent studies have reported dismal outcomes of patients with hepatic‐sided T2 GBC, but the impact of hepatic resection for these patients is not clear.^40^, ^41^

Indication for extrahepatic bile duct resection (EBDR) in cases with GBC that do not have direct invasion to the hepatoduodenal ligament is another major controversy in the surgical treatment of GBC. Kurahara et al^42^ analyzed the impact of EBDR on patient outcomes and showed that EBDR improved outcomes only in patients with proximally located tumors; outcomes were not improved in patients with distally located tumors. In contrast, a different report concluded that EBDR might offer no advantage in long‐term survival for any patient group, including patients with T3 GBC in the EBDR group who required bisectionectomy, hemihepatectomy, trisectionectomy, or combined resection of adjacent organs.^43^ To address this question, prospective RCT including patients with limited disease are needed.

#### 2.2.4 Combined vascular resection

Advances in vascular anastomosis and reconstruction techniques based on transplant surgeries have made combined vascular resection in BTC surgery possible and have contributed to the expansion of surgical indications. Japanese guidelines described that combined portal vein resection (PVR) improves the surgical outcome of patients with portal vein invasion; however, the clinical benefits of combined arterial resection for patients with arterial invasion remain unclear.^32^

##### Combined portal vein resection

Higuchi et al^44^ carried out combined PVR for 69 patients with PHCC. Vascular complications, including portal vein thrombosis, stenosis, and bleeding, occurred in seven cases.^44^ Among 56 patients with PVR, excluding simultaneous hepatic artery resection cases, the mortality rate was 5.4%.^44^ In patients with PVR for portal vein invasion without invasive carcinoma in the ductal margin or distant metastases, 5‐year OS was 35.6%, which was significantly worse than that of patients with no portal vein invasion (53.4%); however, patients with portal vein invasion and distant metastasis or invasive carcinoma in the ductal margin had a 5‐year OS of 0%.^44^ Molina et al^45^ carried out combined PVR and reconstruction for 23 patients with PHCC; among these, five patients (22%) died after surgery, but portal vein invasion did not affect DSS or OS. A report from a multi‐institutional database from the USA also showed favorable outcomes of combined PVR.^46^

Outcome of combined PVR and/or IVC resection for patients with ICC was reported in a recent study of 128 patients.^47^ Although severe complications occurred in 26.6% of these patients and 90‐day mortality rate was 7.0%, these outcomes were similar to those of patients without vascular resection;^47^ additionally, long‐term outcomes were similar among patients with and without combined vascular resection.^47^ In contrast, the Nagoya group reported that PVR for distal cholangiocarcinoma (DCC) did not contribute to long‐term survival.^8^ The authors concluded that DCC with suspected PV invasion may be categorized as a borderline resectable tumor, because PV invasion in DCC was a negative prognostic factor with high T classification, lymphatic invasion, perineural invasion, pancreatic invasion, and LN metastasis in that study.^8^

##### Combined artery resection

Higuchi et al^44^ reported the outcomes of combined hepatic artery resection for 19 patients with PHCC in 2018; vascular complication occurred in five cases (26%), which included pseudoaneurysm, obstruction of the reconstructed artery, and intra‐abdominal bleeding, whereas in‐hospital death occurred in three cases. The 5‐year OS of patients with hepatic artery invasion was 24.7%, which was significantly worse than that of patients without hepatic artery invasion (53.4%) among patients without distant metastasis or invasive carcinoma in the ductal margin.^44^ However, the 5‐year OS of patients with hepatic artery invasion with distant metastasis or invasive carcinoma in the ductal margin was 0%.^44^ The previously mentioned report from a multi‐institutional US database also showed favorable outcomes of combined hepatic artery resection.^46^

In contrast, the Shizuoka Cancer Center group in Japan reported major hepatectomy for 29 patients with advanced GBC, including six patients who underwent combined artery resection.^9^ These authors concluded that GBC with hepatic artery invasion and liver metastasis were contraindications to surgery because the outcomes of patients with GBC associated with hepatic artery invasion or liver metastasis were comparable to those of patients with unresectable disease.^9^ Similarly, the outcome of patients who underwent PD with combined venous and arterial resection for PC was similar to that of patients who underwent palliative bypass.^48^

#### 2.2.5 Laparoscopic approach

A meta‐analysis comparing the laparoscopic (Lap) and the open (Op) approach for GBC showed superior 5‐year survival and postoperative outcomes in Lap compared to Op; however, the scar recurrence rate was significantly higher in Lap than in Op.^49^ Some authors reported the safety and efficacy of laparoscopic radical cholecystectomy, including whole‐layer cholecystectomy, gallbladder bed resection, or segment IVb/V resection with LND for gallbladder lesions suspected to be GBC or incidental GBC.^50^, ^51^, ^52^ Further investigation is needed to elucidate the optimal strategy for gallbladder lesions suspected to be GBC.

A recent report showed acceptable short‐ and long‐term outcomes of patients with large (>5 cm) or multiple ICC who underwent laparoscopic liver resection.^53^ Some authors reported that laparoscopic lymphadenectomy for patients with ICC and GBC was safe^54^, ^55^ and feasible for retrieving sufficient LN,^55^ whereas a different study reported that LN yield by Lap was significantly lower than that by Op.^56^

Corresponding to rapid technological progress, robot‐assisted laparoscopic surgery has been gradually applied for HBP surgery worldwide. Liu et al^57^ reported the surgical outcomes of robot‐assisted laparoscopic pancreaticoduodenectomy compared to those of the conventional laparoscopic approach. Although further investigations of the usefulness and cost‐effectiveness of surgical robot systems are needed, such systems equipped with multi‐joint forceps could be suitable for HBP surgeries, which require complex techniques and highly refined skills.

### 2.3 Repeat resection for recurrent cholangiocarcinoma

Although the mainstream treatment for recurrent cholangiocarcinoma is systemic chemotherapy, the survival benefits of aggressive surgical resection for recurrence have been recently reported. Yamashita et al^58^ reported good overall survival of surgical treatment for ICC recurrence with a 5‐year survival rate of 44%, which was comparable to that after primary surgery for ICC. As outcomes after the second operation for patients with intrahepatic metastasis in primary surgery were worse than in those without primary intrahepatic metastasis, the authors concluded that repeat resection for ICC recurrence with primary intrahepatic metastasis should be considered as a contraindication.^58^ Kyoto University group also reported the outcomes of repeat resection for recurrent ICC.^59^ In that study, in intrahepatic recurrent (MST after recurrence: not reached vs 8.9 months, *P* < 0.001) and extrahepatic recurrent (MST after recurrence: 80.4 months vs 11.7 months, *P* < 0.001) subgroups, outcomes of patients who underwent repeat resection had better outcomes than those who did not.^59^ Multivariate analysis to confirm prognostic factors after recurrence in 108 patients with or without repeat resection showed that repeat surgery, time to recurrence longer than 1 year, and gemcitabine (GEM)‐based systemic chemotherapy were independent prognostic factors.^59^ In a study published by the Nagoya University group, outcomes of patients with recurrent pulmonary metastasis of cholangiocarcinoma who underwent resection were better than in those without resection, and multivariate analysis to identify prognostic factors after intrapulmonary recurrence showed that time to recurrence longer than 2 years and resection of pulmonary metastasis were prognostic factors.^60^ Based on the findings of these studies, active resection of the recurrence site could be effective in cases with limited recurrent disease and relatively longer time to recurrence.

### 2.4 Perioperative management

#### 2.4.1 Perioperative prophylactic antibiotics

Biliary tract cancer frequently causes bile duct obstruction which requires preoperative biliary drainage (PBD). Patients undergoing resection after PBD are at high risk of surgical site infection (SSI) and perioperative antibiotic prophylaxis is widely used with various antibiotic regimens and durations. A RCT to evaluate the optimal duration of giving prophylactic antibiotics for patients undergoing major hepatectomy with EBDR found similar incidence rates of infectious complications, additional antibiotic use, and grade IIIa or higher complications, whereas postoperative hospital stay did not differ between a 2‐day or a 4‐day administration group without restriction of antibiotic regimen.^10^

The optimal duration of prophylactic antibiotics for patients undergoing PD after PBD was investigated in a recent RCT in which patients were randomized to groups of 1‐day or 5‐day administration of cefozopran.^20^ Incidence of overall infectious complications, intra‐abdominal abscess, clinically relevant postoperative pancreatic fistula, and Clavien‐Dindo grade III or higher complications was lower in the 1‐day group, and duration of postoperative hospital stay was also shorter in the 1‐day group.^20^ Although further investigation of the optimal selection and duration of prophylactic antibiotics is needed, shortening the duration of prophylaxis may be feasible and desirable.

#### 2.4.2 Preoperative biliary drainage

The Japanese guidelines recommend endoscopic nasobiliary drainage (ENBD) for its low risk of complications.^32^ According to a report from the Nagoya University group, percutaneous transhepatic biliary drainage (PTBD) was an independent predictor of poorer survival of patients with resectable PHCC and a risk factor for seeding metastasis, including PTBD catheter tract recurrence, peritoneal dissemination, and pleural dissemination.^61^ The outcomes of patients who underwent PTBD were significantly worse than those of patients with endoscopic biliary drainage (EBD) after PSM.^61^ Pleural dissemination of cholangiocarcinoma caused by PTBD was also reported from the same institute: 12 of 212 patients who underwent right‐sided PTBD developed pleural dissemination on the right side of the thoracic cavity after resection with a median time of 381 days after resection.^62^ In contrast, a different study from the USA reported that DSS and RFS after resection were similar between patients who underwent PTBD and EBD.^63^ A prospective RCT is needed to elucidate whether PTBD or EBD is a more optimal treatment; however, a multicenter prospective RCT in the Netherlands was prematurely closed because of drastically higher mortality in the PTBD group (41%) compared to the EBD group (which was also notably high, at 11%).^21^

#### 2.4.3 Advantages and disadvantages of transfusion

Although some authors reported that perioperative transfusion was associated with poorer survival after resection,^23^ other studies using PSM have shown that perioperative transfusion did not affect long‐term outcomes.^64^, ^65^ In contrast, perioperative transfusion was found to be associated with an increased risk of postoperative complications.^23^, ^64^

Onoe et al. compared the outcomes of patients who underwent resection with deposited autologous blood transfusion with those of patients who underwent resection with homologous blood transfusion and found that, although postoperative maximum total bilirubin level was significantly lower in the autologous group, the incidence of major complications, including liver failure, mortality, and long‐term outcomes, was similar between the two groups after PSM.^66^ In general, homologous blood transfusion should be avoided when possible, considering transfusion‐related complications and limited sources of homologous blood; autologous transfusion could be an option.

#### 2.4.4 Prediction of postoperative liver failure and optimization of future liver remnant

Portal vein embolization (PVE) is a breakthrough technique to optimize the future liver remnant (FLR) and reduce operative mortality. The Japanese guidelines recommend that preoperative PVE should be considered for patients scheduled to undergo surgical resection combined with right hepatectomy or more, or hepatectomy of ≥50‐60% of liver volume.^32^ The Academic Medical Center group in Amsterdam, The Netherlands, reported that the indication for PVE based only on remnant liver volume is insufficient, and other predictors of liver failure should be considered.^67^ According to the authors of that report, if a patient had two of three predictors, including jaundice at presentation, preoperative cholangitis, and immediate preoperative bilirubin higher than 2.9 mg/dL, the predicted risk of liver failure after surgery was 44% even if FLR was ≥45%.^67^ The same group reported the value of a preoperative assessment of FLR function using ^99mTc^‐mebrofenin hepatobiliary scintigraphy in patients with PHCC to predict postoperative liver failure.^68^

A group from Chiba University in Japan reported that the incidence of severe postoperative complications after major hepatectomy with EBDR in patients older than 65 years was higher than that in younger patients.^11^ The authors also reported that delayed liver regeneration was the reason for the age‐related risk, and that the incidence of severe postoperative complications in older patients significantly decreased if the percentage of the FLR volume was set at ≥45%.^11^ Based on these findings, the authors suggested that preoperative PVE should be considered in patients older than 65 years undergoing major hepatectomy with EBDR with % FLR volume <45%, and reported that increasing rates of FLR volume after PVE were similar between younger and older patients.^11^

According to a report from the University of Tokyo, there was no difference in the degree of hypertrophy among cancer types, including hepatocellular carcinoma (HCC), BTC, and colorectal liver metastases (CLM), despite differences in liver function and background liver diseases.^69^ Among 319 patients, complications associated with PVE occurred in 25 (7.8%) patients; these included portal vein thrombosis, bleeding, bile leak, bowel obstruction, and coil misplacement, and one patient died at post‐PVE day 57 from massive subcapsular hemorrhage.^69^ The dropout rate after PVE was higher in patients with BTC or CLM, mainly because of disease progression.^69^

Associating liver partition and portal vein ligation for staged hepatectomy (ALPPS) has been introduced as a new technique to induce rapid FLR hypertrophy and provide curative resection for advanced primary or secondary hepatic tumors. However, a recent report from the international ALPPS registry showed that patients with PHCC who underwent resection after ALPPS had a 90‐day mortality rate of 48% and that all patients had a MST of 6 months.^70^ Considering these data, PVE might be recommended over ALPPS to induce FLR hypertrophy for resection in PHCC.

### 2.5 Prediction of recurrence and long‐term outcomes after resection

Several reports have shown that R1 resection, LN metastases, perineural invasion, vascular invasion, and combined vascular resection are prognostic factors after curative‐intent resection. Yamashita et al. reported that non‐normalization of CA19‐9 after curative‐intent resection of BTC was associated with worse OS.^12^ Additionally, a research group from Hannover Medical School in Germany developed a prognostic score using variables available before treatment; these included age, metastasis, C‐reactive protein levels, international normalized ratio, and bilirubin.^71^

Surgical site infection is one of the most common complications after HBP surgery. Multivariate analysis of data from a large, multicenter database in the USA showed that in patients undergoing resection of extrahepatic BTC, SSI adversely affected the long‐term outcomes of patients in an entire cohort (n = 728) as well as in a subgroup (n = 279) of patients with well‐to‐moderate tumor differentiation, R0 resection, and no LN metastasis.^72^ Studies on the prognostic factors of each cancer type are shown below.

#### 2.5.1 Intrahepatic cholangiocarcinoma

A recent study analyzed the impact of morphological status on long‐term outcomes after curative‐intent resection using a large multi‐institutional international cohort.^73^ Results showed that patients with periductal‐infiltrating‐type or mass‐forming + periductal‐infiltrating‐type ICC had an approximately 45% increased long‐term risk of death compared to patients with mass‐forming or intraductal‐growth‐type ICC.^73^

Another problem is the confusing difference between PHCC and ICC involving hepatic hilum (hICC). Using a large multi‐institutional international database, Zhang et al^74^ analyzed differences in the clinicopathological features and short‐ and long‐term outcomes of PHCC and hICC defined by Ebata et al.^75^ In that study, hICC had a more aggressive phenotype and a higher frequency of vascular invasion and LN metastases, and needed more extensive resection compared with PHCC and peripheral ICC.^74^ More technical complications were reported and long‐term outcomes were worse in patients with hICC compared to those with PHCC and peripheral ICC.^74^

Sarcopenia is another condition that affects the surgical outcome of patients with several types of cancer. Okumura et al^76^ reported that low skeletal muscle mass and quality were associated with poorer long‐term outcome of patients with stage I‐III ICC.

Following curative‐intent resection for ICC, many prognostic factors have been reported, including age,^13^ positivity for hepatitis B surface antigen,^77^ neutrophil‐to‐lymphocyte ratio,^78^, ^79^ C‐reactive protein,^79^ albumin,^78^ CA19‐9,^78^, ^79^ tumor size,^58^, ^77^, ^78^ multifocal tumor,^13^, ^14^ extrahepatic extension,^13^ intrahepatic metastasis,^58^ Child‐Pugh score,^77^ LN metastasis,^13^, ^58^, ^77^ pathological lymphatic infiltration,^58^ pathological bile duct invasion,^58^ and non‐curative resection.^14^ A recent report showed that tumor size and number of tumors were associated with early (<24 months) intrahepatic recurrence and only the presence of liver cirrhosis was associated with late intrahepatic recurrence (>24 months).^15^

#### 2.5.2 Perihilar cholangiocarcinoma

Ebata et al^2^ reported that PTBD, blood transfusion, LN metastasis and distant metastasis were prognostic factors of long‐term survival, whereas vascular resection was not. van Vugt et al^80^ reported that unilateral and main/bilateral hepatic artery involvement was a poor prognostic factor, whereas portal vein involvement was not.

#### 2.5.3 Distal cholangiocarcinoma

Preoperative biliary stenting,^81^ extent of surgery in cases of positive histological venous invasion,^81^ perineural invasion,^16^, ^81^ pancreatic invasion,^16^ positive resection margin,^82^ high tumor grade,^82^ LN metastasis,^16^, ^81^, ^82^ distant metastasis,^82^ and postoperative complications^81^, ^82^ were reported as prognostic factors after resection.

#### 2.5.4 Gallbladder cancer

Ethun et al^83^ used the data of 449 patients with incidental GBC to develop the GBC predictive risk score (GBRS) which comprises T stage, differentiation grade of the tumor, and presence of lymphovascular and perineural invasion. GBRS was associated with increased incidence of locoregional residual and distant disease at reoperation and worse OS.^83^ Mochizuki et al^84^ also reported that high GBRS was a prognostic factor after curative resection. A recent report described a preoperative prediction model of incidental GBC that included age, female gender, previous cholecystitis, and the combination of acute cholecystitis without jaundice or jaundice without acute cholecystitis; predictive ability was improved with macroscopic evaluation of the gallbladder.^85^ Other prognostic factors of GBC after resection included resection margin status,^86^ TNM stage,^86^ albumin level,^86^ fibrinogen,^87^ CA19‐9,^87^ and fibrinogen‐to‐albumin ratio.^86^

#### 2.5.5 Ampullary cancer

In patients with ampullary cancer, age,^88^, ^89^ positive resection margin,^89^ <12 retrieved LN,^89^ LN ratio,^90^ pancreatobiliary subtype,^88^ and elevated preoperative carcinoembryonic antigen (CEA)^88^ were reported as prognostic factors after resection. A nomogram composed of AJCC pathological T and N classification, histological differentiation, lymphovascular invasion, and perineural invasion for predicting the probability of recurrence after resection was also reported.^91^

### 2.6 Liver transplantation for cholangiocarcinoma

Although liver transplantation (LT) for HCC has been proven as a beneficial option for patients selected with the Milan criteria, cholangiocarcinoma could be a contraindication for LT as a result of its high recurrence rate and poor long‐term outcomes. However, vigorous attempts have been made to improve the outcome of patients with BTC using neoadjuvant therapy (NAT) under strict inclusion criteria and, under these inclusion criteria, it is time to reconsider the indications for LT in patients with BTC. According to a recent report using a retrospective collaborative database from 10 academic institutions in the USA, LT for tumors <3 cm with LN‐negative disease in patients without primary sclerosing cholangitis was associated with improved OS compared with resection (3‐year: 54% vs 44%, 5‐year: 54% vs 29%; *P* = 0.03).^17^ However, this study had some limitations: the short‐ and long‐term outcomes of the resection group were very poor, and the good candidates for LT proposed by the authors were very limited cases: “solitary, <3 cm in diameter, no LN metastasis, but unresectable.”^92^ The definition of “unresectable disease” is not universal, and some centers resect tumors diagnosed as “unresectable” in another center. Moreover, NAT was carried out in only 5% of the resection group compared to 95% of patients in the LT group, and it is unclear whether sufficient efforts were made to improve the surgical outcome of resection.

The Mayo and Cleveland Clinic research group reported the outcomes of LT for patients who were initially diagnosed with HCC and subsequently found to have either ICC or combined HCC‐cholangiocarcinoma on explant.^93^ Higher recurrence rates and worse survival were observed even in cases of early ICC or combined HCC‐cholangiocarcinoma, which was defined as a single lesion <2 cm, compared with HCC.^93^ Lunsford et al^94^ described the relatively good outcome of LT for six selected patients with locally advanced and unresectable ICC in a noncirrhotic liver after confirmed disease stability following 6 months of NAT that consisted of GEM‐based chemotherapy. Considering the chronic shortage of grafts from deceased and living donors, careful consideration and discussion are needed prior to introducing LT for cholangiocarcinoma.

## 3 CHEMOTHERAPY AND RADIATION FOR BILIARY TRACT CANCER

Although GEM plus cisplatin is standard care for unresectable and metastatic BTC, the efficacy of novel doublet (GEM plus S‐1) or triplet (GEM plus cisplatin plus S‐1) chemotherapies has also been reported. The checkpoint inhibitors that include pembrolizumab are also promising, especially for patients with microsatellite‐unstable BTC. Moreover, next‐generation sequencing analysis has also identified actionable mutations, including fibroblast growth factor receptor fusion rearrangement, and isocitrate dehydrogenase‐1 and ‐2 mutations, and clinical trials with targeted drugs have begun.^95^ These regimens will be evaluated in pre‐ or postoperative adjuvant settings in the near future. In this article, we overview published data with a special focus on perioperative therapies.

### 3.1 Adjuvant chemotherapy

The outcomes of patients with node‐positive BTC cannot be improved by surgery alone,^96^ and several retrospective studies have reported the value of adjuvant chemotherapy especially in LN‐positive BTC;^97^, ^98^, ^99^ however, the optimal regimen has not been established. A recent Japanese RCT evaluating the effect of adjuvant chemotherapy with GEM for resected BTC failed to show a significant difference in OS or RFS between the GEM and the observation groups.^18^ However, the BILCAP trial in the UK showed a longer OS in a capecitabine (Cape) group versus an observation group in per‐protocol analysis only (51 vs 36 months; *P* = 0.028; Primrose JN, 2017, unpublished data). Several small studies showed promising results of adjuvant chemotherapy with S‐1^19^, ^100^, ^101^ or GEM plus cisplatin^102^ or GEM plus cisplatin plus 5‐fluorouracil.^103^ Several RCT of adjuvant chemotherapy for resected BTC are ongoing; for example, PRODIGE12‐ACCORD 18 (GEM plus oxaliplatin; Edeline J, 2017, unpublished data), ASCOT (S‐1),^104^ ACTICCA‐1 (NCT02170090; GEM plus cisplatin), NCT02798510 (GEM + cape + radiation vs GEM + cape), and NCT02548195 (GEMOX vs Cape).

### 3.2 Neoadjuvant chemotherapy and chemoradiation therapy

Kobayashi et al^105^ investigated the impact of neoadjuvant chemoradiation therapy with full‐dose GEM and radiation, and reported that the RFS of patients who received NAT was better than that of patients without (3‐year RFS: 78% vs 58%, *P* = 0.0263). Although crude OS was similar between the groups, OS adjusted by inverse probability of treatment weighting was improved by NAT (*P* = 0.00187).^105^ Future studies should clarify the indications for NAT and develop an effective regimen.^106^

## 4 CONCLUSION

We reviewed current trends in the surgical treatment of BTC. Advanced cholangiocarcinoma remains an intractable disease with dismal prognosis, requiring a multidisciplinary approach. There are many problems that should continue to be investigated, including safety and significance of combined hepatic artery resection in PHCC, accurate preoperative diagnosis of GBC and LN metastasis, NAT and adjuvant treatment for advanced disease, and the indication and strategy of LT for BTC. Future research is required in these areas to improve the clinical outcomes of patients with BTC.

## DISCLOSURE

Conflicts of Interest: Authors declare no conflicts of interest for this article.

Author Contribution: Hiroaki Nagano devised the project, the main conceptual ideas and proof outline. Satoshi Matsukuma selected and reviewed references, wrote the initial draft of the manuscript. Yukio Tokumitsu, Yoshitaro Shindo and Hiroto Matsui contributed to review references and assisted in the presentation of the manuscript. All authors critically reviewed the manuscript.
